# Functional characterization of *BRCC3* mutations in acute myeloid leukemia with t(8;21)(q22;q22.1)

**DOI:** 10.1038/s41375-019-0578-6

**Published:** 2019-10-01

**Authors:** Tatjana Meyer, Nikolaus Jahn, Stefanie Lindner, Linda Röhner, Anna Dolnik, Daniela Weber, Annika Scheffold, Simon Köpff, Peter Paschka, Verena I. Gaidzik, Dirk Heckl, Sebastian Wiese, Benjamin L. Ebert, Hartmut Döhner, Lars Bullinger, Konstanze Döhner, Jan Krönke

**Affiliations:** 1 Department of Internal Medicine III, University Hospital of Ulm, Ulm, Germany; 2 Department of Hematology, Oncology, and Tumorimmunology, Charité University Medicine, Berlin, Germany; 3 Department of Pediatric Hematology and Oncology, Martin-Luther-University Halle-Wittenberg, Halle, Germany; 4 Department of Medical Oncology, Dana-Farber Cancer Institute, Boston, MA USA

**Keywords:** Acute myeloid leukaemia, Translational research

## Abstract

*BRCA1/BRCA2-containing complex 3* (*BRCC3*) is a Lysine 63-specific deubiquitinating enzyme (DUB) involved in inflammasome activity, interferon signaling, and DNA damage repair. Recurrent mutations in *BRCC3* have been reported in myelodysplastic syndromes (MDS) but not in de novo AML. In one of our recent studies, we found *BRCC3* mutations selectively in 9/191 (4.7%) cases with t(8;21)(q22;q22.1) AML but not in 160 cases of inv(16)(p13.1q22) AML. Clinically, AML patients with *BRCC3* mutations had an excellent outcome with an event-free survival of 100%. Inactivation of *BRCC3* by CRISPR/Cas9 resulted in improved proliferation in t(8;21)(q22;q22.1) positive AML cell lines and together with expression of *AML1-ETO* induced unlimited self-renewal in mouse hematopoietic progenitor cells in vitro. Mutations in *BRCC3* abrogated its deubiquitinating activity on IFNAR1 resulting in an impaired interferon response and led to diminished inflammasome activity. In addition, *BRCC3* inactivation increased release of several cytokines including G-CSF which enhanced proliferation of AML cell lines with t(8;21)(q22;q22.1). Cell lines and primary mouse cells with inactivation of *BRCC3* had a higher sensitivity to doxorubicin due to an impaired DNA damage response providing a possible explanation for the favorable outcome of *BRCC3* mutated AML patients.

## Introduction

Acute myeloid leukemia (AML) is caused by genetic alterations that lead to enhanced proliferation and a differentiation block in hematopoietic progenitor cells. Although remissions can be achieved in the majority of patients with intensive chemotherapy, a large proportion of patients relapse which is associated with impaired survival. Recurrent genomic changes found in AML include structural chromosomal aberrations, translocations, and gene mutations. The most frequently found translocations/inversions are t(8;21)(q22;q22.1) and inv(16)(p13.1q22) or t(16;16)(p13.1q22) that together form the subgroup of core-binding factor (CBF) AML [1–4]. CBF is a heterodimeric transcription factor complex comprising RUNX1 and CBFB that is essential for the expression of genes vital for normal hematopoiesis. Lack or mutation of one of the complex members (RUNX1 in t(8;21)(q22;q22.1) AML or CBFB in inv(16)(p13.1q22) AML) results in a dominant negative inhibitory effect on CBF complex formation and impairment of hematopoiesis [5, 6]. The cytogenetic rearrangement t(8;21)(q22;q22.1) results in the fusion gene *RUNX1-RUNX1T1* (*AML1-ETO*) [1, 2]. While the presence of *AML1-ETO* is considered to impair myeloid differentiation, mouse models suggest that it is not sufficient to induce leukemia [7]. Consistently, almost all AML cases with the t(8;21)(q22;q22.1) abnormality have concurrent genomic aberrations like mutations in *KIT*, *FLT3*, *NRAS*, and *ASXL2* [8, 9]. Although they share similar biological effects like repression of CBF target genes and clinical characteristics including a favorable prognosis [10], t(8;21)(q22;q22.1) and inv(16)(p13.1q22) AML differ in regard to co-occurring mutations. For example, mutations in *ASXL1* and *ASXL2* occur at a high frequency in AML with t(8;21)(q22;q22.1) but are absent in AML with an inv(16)(p13.1q22) implying that they selectively cooperate with *AML1-ETO* [11–13].

BRCA1-/BRCA2-containing complex 3 (BRCC3, also known as BRCC36) is a Lysine-63-specific deubiquitinating enzyme (DUB) and member of the Zn^2+^ dependent JAB1/MPN/Mov34 metalloenzyme (JAMM) domain metalloprotease family [14, 15]. In contrast to ubiquitin chains linked at other another lysine residue, lysine-63 ubiquitination does in general not lead to proteasomal degradation of the target protein but instead to altered protein function. BRCC3 is a member of at least two complexes: the nuclear BRCA1-A complex consisting of Abraxas, BABAM1/MERIT40, BABAM2/BRCC45, UIMC1/RAP80, and BRCC3 [16–18], and the cytoplasmic BRCC36 isopeptidase complex (BRISC) including ABRO1, BABAM1, BABAM2, SHMT2, and BRCC3 [19–23]. Following DNA double strand breaks (DSBs), the E3 ubiquitin ligase RNF8 specifically adds K63-ubiquitin to histones H2A and H2AX [24], which in turn get recognized by the tandem ubiquitin interacting motif (UIM) of UIMC1 [25]. This leads UIMC1 to guide the BRCA1-A complex towards the DSB for DNA repair. BRCC3 finally resolves the interaction of the BRCA1-A complex with the DNA by cleaving off the K63-ubiquitin chains from H2A and H2AX [16]. Accordingly, mutations or deletions of *BRCC3* have been shown to result in impaired DNA damage repair [26]. As a member of the BRISC, BRCC3 is involved in deubiquitination and thus regulation of different targeting proteins, including NLR family pyrin domain containing 3 (NLRP3) and type 1 interferon (IFN) receptor chain 1 (IFNAR1) [20, 27]. Loss of *BRCC3* has been linked to decreased release of mature interleukin (IL)-1β by dysregulation of NLRP3 and the inflammasome [27] while impaired deubiquitination of IFNAR1 is associated with diminished induction of interferon signaling [20].

While recurrent mutations in *BRCC3* have been recently described in myelodysplastic syndromes (MDS) [28], only one *BRCC3* mutation has been identified in a patient with complex karyotype in the TCGA AML cohort of 200 cases [29]. In one of our sequencing studies of a large cohort of CBF AML patients, we exclusively found recurrent *BRCC3* mutations in t(8;21)(q22;q22.1) AML [30]. Here, we investigated the impact of *BRCC3* mutations on malignant transformation and drug sensitivity in human and murine hematopoietic cells.

## Materials and methods

### Patients

The targeted sequencing study included 351 CBF AML cases [30] with a t(8;21)(q22;q22.1) (*n* = 190) or inv(16)(p13.1q22) (*n* = 161). Genetic analyses were approved by the local ethics committee and all patients provided informed consent in accordance with the declaration of Helsinki. Further details about the patients and treatment are given in the supplementary information.

### Plasmids

The following plasmids were previously described: pLKO5d.SFFV.SpCas9.P2A.BSD, pLKO5.sgRNA.EFS.PAC, pLKO5.hU6.sgRNA.dTom, and pRSF91-MCS-IRES-GFP-T2A-Puro [31]. pMY-IRES-GFP and pMY-IRES-GFP-AML1/ETO were kindly provided by F. Oswald (Internal Medicine I, University Hospital of Ulm). IFNAR1_pCSdest was a gift from Roger Reeves (Addgene plasmid # 53881) [32].

### Genetic inactivation by CRISPR/Cas9 in cell lines

Kasumi-1, SKNO-1, THP-1, OCI-AML5, HEK-293T, and 32D cell lines stably expressing the Cas9 enzyme were generated using the pLKO5d.SFFV.SpCas9.P2A.BSD lentiviral vector and selected with blasticidin (10 µg/ml) (ant-bl, InvivoGen, San Diego, CA, USA). Presence of the FLAG-tagged Cas9 enzyme was confirmed by western blot. When generating a *BRCC3* or *Brcc3* knockout, vectors containing one or multiple sgRNA against *BRCC3* or *Brcc3* were introduced into the cell using the pLKO5.sgRNA.EFS.PAC plasmid and selected with puromycin (2 µg/ml) (ant-pr-1, InvivoGen). The following sgRNA-sequences were used: *BRCC3* #1: AGCGTGGTTGAGACAAACG; *BRCC3* #2: TCTAGTTGAACGATGATACA; *Brcc3* #1: GGAGGTAAGTTGGCCACCT; *Brcc3* #2: TGTGTATAGGGGAGGTAAGT; *Brcc3* #3: TGTCATCATTCAACTAGAGT; *Luciferase* control: AGTTCACCGGCGTCATCGTC. The knockout was confirmed by western blot and Sanger sequencing.

### Western blot

Protein concentrations were determined using the BCA Protein Assay Kit (23225, Thermo Fisher Scientific). The following antibodies were used: BRCC3: ab108411, Abcam, Cambridge, UK; Tubulin: T5168, Sigma-Aldrich; ABRO1: ab74333, Abcam; Abraxas: ab139191, Abcam; BABAM1: sc-160990, Santa Cruz, Dallas, TX, USA; UIMC1: 14466S, New England Biolabs, Ipswich, MA, USA; FLAG-tag (DYKDDDDK): 2368, Cell Signaling Technology, Danvers, MA, USA; HA-tag (YPYDVPDYA): 901533, BioLegend; G-CSF: sc-53292, Santa Cruz; β-Actin HRP: ab20272, Abcam; secondary anti-mouse HRP: 7076S, Cell Signaling; secondary anti-rabbit HRP: 7074S, Cell Signaling.

### DNA damage and apoptosis

To assess DNA damage, Kasumi-1 *BRCC3* WT, R81X, R81G, or KO cells were cultured at a density of 1 × 10^6^ cells and treated with different concentrations of doxorubicin (S1208, SelleckChem, Houston, TX, USA) or DMSO as a vehicle control for 3 days. The cells were then fixated in ice-cold methanol overnight, stained with an anti-phospho histone H2A.X antibody (05-636-AF647, Merck Millipore) and analyzed using flow cytometry.

To measure the percentage of apoptotic cells in Kasumi-1 *BRCC3* WT and KO cells, 1 × 10^6^ cells were seeded and treated with different concentrations of doxorubicin or DMSO as a vehicle control for 3 days. Then, apoptosis was measured using the FITC Annexin V Apoptosis Detection Kit with 7-AAD (640922, BioLegend, San Diego, CA, USA) according to the manufacturer’s protocol.

### Statistics

Differences between groups were analyzed by an unpaired *t*-test. For contingency tables, a Fisher’s exact test was applied. Kaplan–Meier plots were generated for each time-to-event outcome measure and differences between two groups were analyzed using the two-sided log-rank test. *P* values are displayed as follows: n.s., not significant; *P < 0.05; **P < 0.01; ***P < 0.001. All statistical analyses were performed using Prism version 7 (GraphPad Software, San Diego, CA, USA).

A description of further methods is given in the Supplementary information.

## Results

### *BRCC3* mutations in AML with t(8;21)(q22;q22.1)

We first discovered *BRCC3* mutations in two AML cases (Table 1, patients number #1 and #2) by exome sequencing (data unpublished). Since both *BRCC3* mutated patients had a t(8;21)(q22;q22.1) and no *BRCC3* mutations were found in other types of AML we included *BRCC3* in a targeted sequencing study with a total of 351 patients with core-binding-factor AML including 191 patients with t(8;21)(q22;q22.1) and 160 patients with inv(16)(p13.1q22) [30]. Here, an additional seven *BRCC3* mutations were found for a total of nine (4.7%) mutations in cases with t(8;21)(q22;q22.1) but none were found in the patient cohort with inv(16)(p13.1q22) (Fig. 1a) [30]. Three of the nine mutations were nonsense mutations, two frameshift mutations, three missense mutations and one mutation affected a splice site (Fig. 1b, Table 1). Seven of the nine mutations were located in the catalytically important MPN domain. Using the *in silico* prediction tool Polymorphism Phenotyping v2 (PolyPhen-2) algorithm [33], the coding mutations were all predicted to be damaging to the function of BRCC3. Four of the mutations affected a codon that was previously found to be mutated in MDS [28]. The *BRCC3* gene is located on chromosome Xq28 and *BRCC3* mutations were thus hemizygous in six male and two female patients with loss of one of the X chromosomes. However, based on the variant-allele fraction the majority of *BRCC3* mutations were subclonal (Table 1).

**Table 1:** *BRCC3* mutations found in t(8;21)(q22;q22.1) AML

#	Age	Karyotype	*BRCC3* mutation		VAF	BM blasts (%)
1	27	45,X,-Y,t(8;21)(q22;q22.1),del(9)(q13q22)[20]	C265T	R89X	0.90	70
2	44	46,XY,t(8;21)(q22;q22.1),del(9)(q13q22)[18]/ 45,X,-Y,t(8;21)(q22;q22.1),del(9)(q13q22)[4]	C241G	R81G	0.66	60
3	19	46,XY,t(8;21)(q22;q22.1)[1]/45,idem,-Y[6]/46,XY[2]	Splice site		0.16	n/a
4	67	45,X,-Y,t(8;21)(q22;q22.1)[19]/46,XY[1]	191delA	E64fs	0.10	50
5	49	NA	C904T	Q302X	0.87	73
6	62	46,XY,t(8;21)(q22;q22.1)[17]/45,idem,-Y[3]	A365G	H122R	0.09	34
7	57	45,X,-X,t(8;21)(q22;q22.1), del(9)(q13q22)[15]/46,XX[1]	C241T	R81X	0.06	87
8	69	46,XX,t(8;21)(q22;q22.1)[15]/45,idem,-X[5]	456_457insGGTCC	L152fs	0.28	80
9	18	46,XX,t(8;21)(q22;q22.1)[13]/47,XX,t(8;21)(q22;q22.1), +15[2]	C737T	A246V	0.34	90

**Fig. 1:**
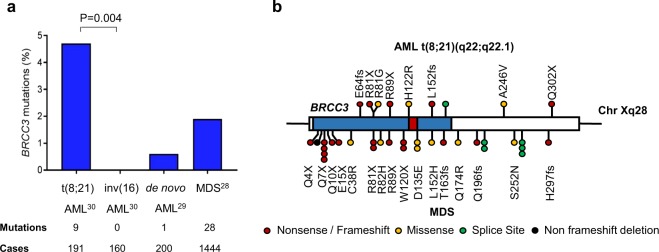
*BRCC3* mutation status in AML and MDS. **a** Frequency of *BRCC3* mutations found in t(8;21)(q22;q22.1) and inv(16)(p13.1q22) AML in 351 patients [30]. Data for MDS and de novo AML was obtained from Huang et al. [28] and the TCGA AML [29] data set, respectively. **b** Overview of the *BRCC3* coding region with mutations analyzed in t(8;21)(q22;q22.1) AML in our study on top and mutations found in Huang et al. and the TCGA data set below. The metalloprotease (MPN) domain is indicated in blue and the JAB1/MPN/Mov34 metalloenzyme (JAMM) domain in red

### *BRCC3* mutations are associated with a favorable prognosis in AML with t(8;21)(q22;q22.1)

**Fig. 2:**
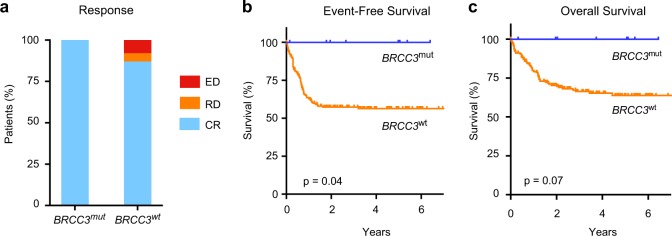
Impact of *BRCC3* mutations on clinical outcome. **a** Response after induction therapy in *BRCC3* mutated and nonmutated patients. CR, complete remission; RD, refractory disease; ED, early death. Kaplan–Maier curves for event-free survival (**b**) and overall survival (**c**) in patients according to *BRCC3* mutation status. Differences between groups were analyzed by a two-sided log-rank test

Correlation with pretreatment clinical data including age, sex, leukocyte count, blood and bone marrow blast count, hemoglobin, platelet counts, or serum LDH levels revealed no significant differences between *BRCC3* mutated and nonmutated patients (Table 2). Also, the number of additional cytogenetic alterations did not differ between *BRCC3* mutated and nonmutated patients. We then investigated whether *BRCC3* mutations affect response and outcome in patients with t(8;21)(q22;q22.1) treated with intensive chemotherapy (Fig. 2). All patients with *BRCC3* mutations achieved a complete remission after induction therapy as compared with 87% of the patients without *BRCC3* mutation (Fig. 2a). None of the patients with a *BRCC3* mutation relapsed resulting in an event-free-survival and overall survival of 100% at 4 years (Fig. 2b, c).

**Table 2:** Clinical characteristics of t(8;21)(q22;q22.1) AML patients with and without *BRCC3* mutations

	*BRCC3*^*mut*^	*BRCC3*^*wt*^	*P*-value
	(*n* = 9)	(*n* = 182)	
Male	6 (67%)	102 (56%)	0.5
Leuk (G/L)	9.1	8.5	0.3
Hb (g/dl)	8.9	8.8	0.9
Thr (G/L)	40	30	0.7
LDH (U/ml)	577	495	0.6
BM blasts (%)	68	60	0.6
Blood blasts (%)	39	37	0.9
Additional cytogenetic alterations			
Any	7 (78%)	108 (59%)	0.3
−X/Y	7 (78%)	78 (43%)	0.1
One X Chr (male/female with -X)	8 (88%)	131 (72%)	0.4
del9q	3 (33%)	24 (13%)	0.1
−7/del7q	0 (0%)	5 (3%)	1.0
+8	0 (0%)	16 (3%)	1.0

In order to determine whether the transcriptional level of *BRCC3* has an impact on outcome in AML, we analyzed the TCGA de novo AML data set [29] with the BloodSpot online tool [34]. Here, patients with low *BRCC3* levels had a better overall survival than patients with high *BRCC3* levels (Supplementary Fig. S1).

### Knockout of *BRCC3* leads to increased cell proliferation in t(8;21)(q22;q22.1) AML cells

**Fig. 3:**
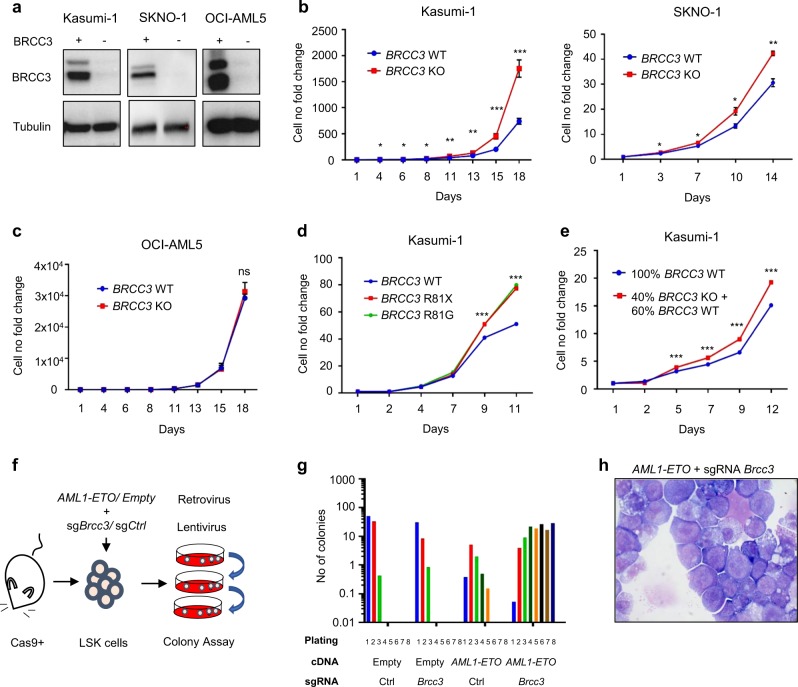
Impact of *BRCC3* mutations on proliferation and self-renewal in vitro. **a** Validation of *BRCC3* knockout via CRISPR/Cas9 in Kasumi-1, SKNO-1, and OCI-AML5 cell lines using western blot. Growth curve of Kasumi-1, SKNO-1, (**b**) and OCI-AML5 (**c**) cells containing either *BRCC3* wild type (WT) or knockout (KO) for a duration of 14 or 18 days. **d** Growth curve of *BRCC3* R81X and R81G mutations in Kasumi-1 cells compared with *BRCC3* WT cells over a duration of 11 days. **e** Growth curve of *BRCC3* KO cells at a subclonal level (40% *BRCC3* KO + 60% *BRCC3* WT) in Kasumi-1 cells over a duration of 12 days. **f** Schematic overview of colony forming unit (CFU) assay using sorted murine hematopoietic stem- and progenitor cells (LSK cells) infected with a retrovirus expressing *AML1-ETO* or a control and a lentivirus expressing a pool of three different sgRNAs targeting *Brcc3* or a non-targeting sgRNA control. After infection, CFUs were seeded for 10–14 days and serially replated. **g** Colony forming unit (CFU) assay. **h** Microscopic assessment of cells taken from the 8th replate of *AML1-ETO* + *Brcc3 KO*. Original magnification ×100. Significance was determined using unpaired Student's *t* test. ^ns^Not significant, *P < 0.05, **P < 0.01, ***P < 0.001. *n* = 3 for data shown, which are representative of three independent experiments

In order to analyze whether loss of *BRCC3* affects cell growth, we determined the effect of CRISPR/Cas9-mediated *BRCC3* inactivation in the two AML cell lines with t(8;21)(q22;q22.1) Kasumi-1 and SKNO-1 and in the OCI-AML5 cell line without t(8;21)(q22;q22.1). Complete knockout (KO) of *BRCC3* was confirmed by western blot (Fig. 3a). We found that *BRCC3* KO resulted in a significantly higher proliferation rate in the two AML cell lines with t(8;21)(q22;q22.1) Kasumi-1 and SKNO-1 (Fig. 3b). In contrast, no differences in proliferation were observed between the OCI-AML5 *BRCC3* WT and KO cells (Fig. 3c). Next, we investigated whether the inactivating *BRCC3* mutations R81X and R81G found in patients with t(8;21)(q22;q22.1) AML had a similar beneficial effect on cell proliferation in Kasumi-1. To this end, we ectopically over-expressed *BRCC3* WT, R81X, or R81G in *BRCC3* KO cells. Again, cell proliferation of Kasumi-1 *BRCC3* R81X and R81G expressing cells was significantly higher as compared with *BRCC3* WT cells suggesting that both mutants are inactivating (Fig. 3d). Lastly, *BRCC3* has been found mutated at a subclonal level in some t(8;21)(q22;q22.1) AML patients. We therefore analyzed the impact of a subclone of *BRCC3* KO on cell proliferation. We found that a fraction of 40% of Kasumi-1 cells harboring a *BRCC3* KO was sufficient to significantly increase cell proliferation of all cells in the culture as compared with *BRCC3* WT cells (Fig. 3e). Of note, the number of cells harboring the *BRCC3* KO remained constant at ~40% implying that the *BRCC3* KO cells stimulated proliferation of *BRCC3* KO and WT cells (Supplementary Fig. S2).

In the next step, we investigated whether loss of *Brcc3*/*BRCC3* is capable of conferring cytokine-independence in murine myeloid 32D or SKNO-1 cells. We found that *Brcc3*-inactivation by CRISPR/Cas9 did not convey IL-3-independent growth in 32D cells as compared with *Kras*^*G13D*^ expression that was used as a positive control (Supplementary Fig. S3a). Likewise, loss of *BRCC3* did not render SKNO-1 cells independent of GM-CSF (Supplementary Fig. S3b). Similarly, inactivation of *Brcc3/BRCC3* did not confer cytokine-independence to Ba/F3 and TF-1 cells (data not shown).

### Loss of *Brcc3* in combination with *AML1-ETO* leads to unlimited self-renewal capacity

In order to investigate the effect of *Brcc3* inactivation on the self-renewal capacity in mouse hematopoietic progenitor cells we performed colony forming assays. To this end, we infected primary murine stem- and progenitor Lineage^-^ Sca1^+^ c-Kit^+^ (LSK) cells isolated from the transgenic Rosa26-Cas9 mouse that constitutively express the Cas9 enzyme [35] with retroviral vectors containing either *AML1-ETO* or a control as well as a lentiviral vector expressing sgRNAs targeting *Brcc3* or a non-targeting sgRNA control (Fig. 3f). While the colony number of cells containing either a control, a single knockout of *Brcc3*, or *AML1-ETO* alone decreased rapidly after 3–5 replatings, cells expressing both, *AML1-ETO* and the *Brcc3*-specific sgRNAs could be replated at least eight times (Fig. 3g). Microscopic assessment of the cells demonstrated an immature blast population in cells transduced with *AML1-ETO* + *Brcc3*^*KO*^ (Fig. 3h) while in the other conditions most cells had a more differentiated phenotype (Supplementary Fig. S4).

### Mutations in *BRCC3* lead to impaired interleukin and interferon signaling

To further analyze the functional impact of *BRCC3* mutations found in AML t(8;21)(q22;q22.1) and MDS patients, we cloned the two missense mutations R81G and R82H, located within the MPN + domain as well as D135E located at the end of the JAMM motif (Fig. 1b). When expressed in HEK-293T cells, all three mutations maintained structural integrity with other BRCA-1A and BRISC complex members at similar levels compared with wild type BRCC3 in a pull-down experiment (Supplementary Fig. S5a). However, both scaffolding proteins Abraxas and ABRO1 were downregulated in Kasumi-1 *BRCC3* KO cells while UIMC1 and BABAM1 were not affected (Supplementary Fig. S5b). In a stable isotope labeling of amino acids in cells (SILAC)-based proteomic interaction partner analysis of FLAG-tagged BRCC3 WT and two recurrent point mutants (R81G and D135E) we did not observe relevant changes in binding to known BRCC3 interactors (Supplementary Fig. S6a–c).

**Fig. 4:**
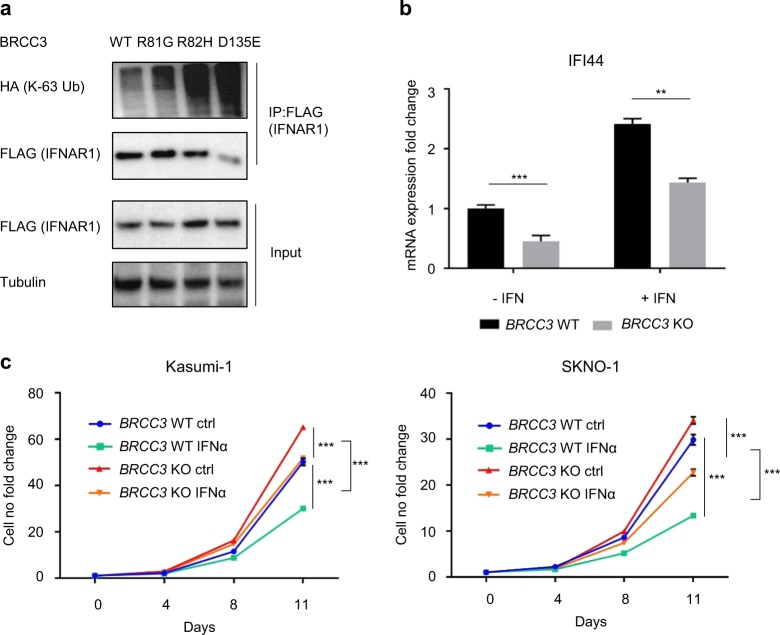
Influence of *BRCC3* mutations on IFNAR1 ubiquitination and IFN signaling. **a** Ubiquitination analysis of IFNAR1 in HEK-293T *BRCC3* KO cells expressing wild-type or mutant *BRCC3*. **b** mRNA expression of the IFN-response gene *IFI44* in Kasumi-1 cells with or without treatment with interferon. **c** Growth curves of *BRCC3* WT or *BRCC3* KO Kasumi-1 and SKNO-1 cells treated with 10 ng/mL IFNα or a vehicle control for a duration of 11 days. ^ns^Not significant, *P < 0.05, **P < 0.01, ***P < 0.001. All *p*-values are calculated using unpaired Student *t* test. Error bars are standard error of the mean. *n* = 2 for Fig. 4a, *n* = 3 for all other experiments, which are representative of two or three independent experiments respectively

Previous studies have identified IFNAR1 and NLRP3 as substrates of the BRCC3 isopeptidase complex (BRISC) [20, 27]. Both proteins rely on deubiquitination mediated by BRCC3 to exercise their cellular functions and shRNA/siRNA mediated knockdown of *BRCC3* has been implicated in impaired activation of IFNAR1 and NLRP3 [20, 27]. NLRP3 is an essential member of the inflammasome that regulates IL-1β. In stimulated THP-1 we found that inactivation of *BRCC3* led to significantly diminished IL-1β release *(*Supplementary Fig. S7a). We then went on to analyze whether addition of IL-1β has a negative effect on proliferation of *BRCC3* WT and KO Kasumi-1 and SKNO-1 cells. While SKNO-1 *BRCC3* WT had a moderately enhanced proliferation in the presence of exogenous IL-1β (Supplementary Fig. S7b), neither SKNO-1 *BRCC3* KO nor Kasumi-1 *BRCC3* WT or KO showed differences in proliferation (Supplementary Fig. S7b, c). Next, we investigated the effect of *BRCC3* mutants on IFNAR1 ubiquitination in *BRCC3* KO HEK-293T cells. K63-linked ubiquitination levels of IFNAR1 were higher in cells expressing *BRCC3* mutants as compared with *BRCC3* WT expressing cells (Fig. 4a). When we checked for interferon response-genes depending on IFNAR1 in Kasumi-1 cells, we observed that upon stimulation with IFN, the induction of IFN response genes *IFI44* (Fig. 4b) as well as of *OASL* (Supplementary Fig. S7d) were diminished in *BRCC3* knockout cells. IFNα has been shown to decrease self-renewal capacity of cells with a t(8;21)(q22;q22.1) background [36]. Consistently, IFNα treatment impaired proliferation of Kasumi and SKNO-1 cells and this effect could be rescued in part by inactivation of *BRCC3* (Fig. 4c).

### Loss of *BRCC3* induces increased cytokine signaling

**Fig. 5:**
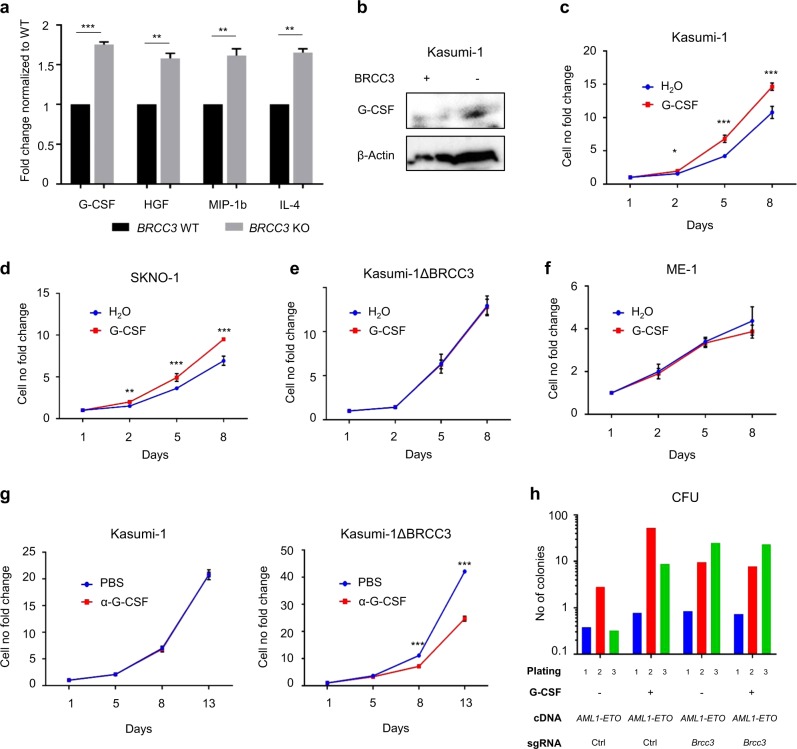
Impact of *BRCC3* inactivation on cytokine release. **a** Top up-regulated cytokines in Kasumi-1 *BRCC3* KO supernatant compared to *BRCC3* WT assessed via Cytokine Array. **b** Western blot depicting G-CSF levels in Kasumi-1 *BRCC3* WT and *BRCC3* KO cells. Growth curves of **c** *BRCC3* WT Kasumi-1 and **d** SKNO-1, **e** *BRCC3* KO Kasumi-1, and **f** *BRCC3* WT ME-1 cells treated with 10 ng/mL G-CSF or a vehicle control for 8 days. **g** Kasumi-1 *BRCC3* WT and *BRCC3* KO cells treated with a neutralizing anti-G-CSF antibody for 13 days. **h** Colony forming unit (CFU) assay of *AML1-ETO* and *AML1-ETO* + *Brcc3* KO cells treated with G-CSF or a vehicle control. ^ns^Not significant, *P < 0.05, **P < 0.01, ***P < 0.001. All *p*-values are calculated using unpaired Student *t* test. Error bars are standard error of the mean. *n* = 2 for Fig. 5h, *n* = 3 for all other experiments for data shown, which are representative of two or three independent experiments respectively

Given the role of BRCC3 in regulation of several cytokines like IL-1β and IFN, we looked for the impact of *BRCC3* inactivation on cytokine release systematically using a cytokine array in unstimulated Kasumi-1 cells. This revealed an upregulation of several cytokines in *BRCC3* KO cells with G-CSF (1.75 ± 0.03) being the top hit (Fig. 5a). Upregulation of G-CSF release was also confirmed by western blot (Fig. 5b). Given the growth stimulating effects of G-CSF on certain AML subtypes including t(8;21)(q22;q22.1) we treated several cell lines with G-CSF. This revealed that cell proliferation of both t(8;21)(q22;q22.1) AML cell lines Kasumi-1 and SKNO-1 with intact *BRCC3* significantly increased when treated with G-CSF (Fig. 5c, d). In contrast, G-CSF did not further enhance the growth of *BRCC3* KO Kasumi-1 cells (Fig. 5e). G-CSF also had no impact on cell proliferation in non-t(8;21)(q22;q22.1) AML cell lines THP (Supplementary Fig. S8) or the inv(16)(p13.1q22) AML cell line ME-1 (Fig. 5f). When treated with a neutralizing anti-GCSF antibody, cell proliferation of Kasumi-1 *BRCC3* KO but not *BRCC3* WT cells decreased (Fig. 5g). Finally, G-CSF increased the colony number of LSK cells transduced with *AML1-ETO* to numbers comparable to cells transduced with *AML1-ETO* + *Brcc3*^*KO*^ which could not be further stimulated (Fig. 5h).

### Inactivation of *BRCC3* results in enhanced sensitivity towards doxorubicin

**Fig. 6:**
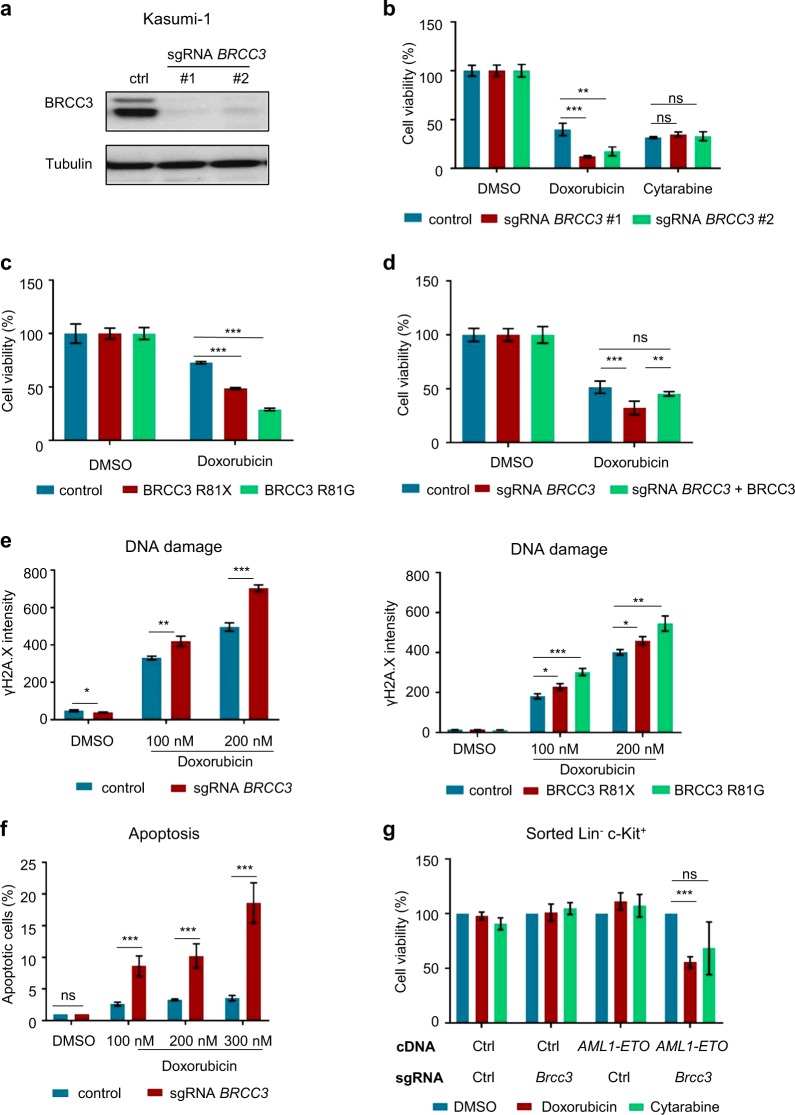
Impact of *BRCC3* inactivation on doxorubicin sensitivity. **a** Confirmation of CRISPR/Cas9 mediated knockout (KO) of *BRCC3* for two different sgRNAs in Kasumi-1 cells by western blot. **b** Cell viability of parental or *BRCC3* KO Kasumi-1 cells after treatment with 7.5 nM doxorubicin or 100 nM cytarabine for 7 days. **c** Cell viability of parental or *BRCC3* R81X and R81G Kasumi-1 cells after treatment with 5 nM doxorubicin. **d** Cell viability of parental, *BRCC3* KO, or *BRCC3* KO ectopically expressing *BRCC3* Kasumi-1 cells after treatment with 5 nM doxorubicin for 7 days. **e** Assessment of phosphorylated γ-H2A.X after treatment with 100 nM or 200 nM doxorubicin or a vehicle control for 3 days in *BRCC3* WT, *BRCC3* R81X, R81G, and KO cells using flow-cytometry. **f** Evaluation of apoptosis in Kasumi-1 *BRCC3* WT or KO cells treated with 100 nM, 200 nM, or 300 nM doxorubicin or a vehicle control for a duration of 3 days. **g** Determination of sensitivity towards doxorubicin and cytarabine in sorted murine hematopoietic stem- and progenitor cells (LSK cells) infected with a retrovirus expressing *AML1-ETO* or empty control and a lentivirus expressing sgRNAs targeting *Brcc3* or a non-targeting sgRNA control. ^ns^Not significant, *P < 0.05, **P < 0.01, ***P < 0.001. All *p*-values are calculated using unpaired Student *t* test. Error bars are standard error of the mean. *n* = 3 for data shown, which is representative of three independent experiments

Downregulation of *BRCC3* has been associated with increased apoptosis in breast cancer cells following ionizing radiation and enhanced sensitivity towards temozolomide in human glioma cells [26, 37]. We investigated whether loss of *BRCC3* renders Kasumi-1 cells more sensitive towards doxorubicin and cytarabine that are the standard treatment for AML with t(8;21)(q22;q22.1). For this purpose, either parental or CRISPR-mediated stable *BRCC3* KO Kasumi-1 cells (Fig. 6a) were treated with doxorubicin for 7 days. We found that knockout with two different sgRNAs targeting *BRCC3* led to a significant decrease in cell viability compared with cells transduced with control sgRNAs (Fig. 6b). Next, the two *BRCC3* mutations R81X and R81G were ectopically over-expressed in *BRCC3* KO cells. Like *BRCC3* KO cells, both *BRCC3* mutants also displayed an enhanced sensitivity towards doxorubicin (Fig. 6c). This enhanced sensitivity to doxorubicin was abrogated when we reintroduced *BRCC3* WT by retroviral expression in *BRCC3* KO cells (Fig. 6d). In contrast to doxorubicin, the sensitivity towards cytarabine was not affected (Fig. 6b). When we checked for DNA damage accumulation caused by doxorubicin treatment, we found that *BRCC3* KO as well as *BRCC3* R81X and R81G mutated cells accumulated significantly more DNA damage as measured by the level of phosphorylated γH2A.X (Fig. 6e). Similarly, the percentage of apoptotic cells was also significantly higher in *BRCC3* KO cells post doxorubicin treatment (Fig. 6f). Lastly, we analyzed mouse LSK cells infected with either *AML1-ETO* or an empty control vector as well as sgRNAs targeting *Brcc3* or a non-targeting sgRNA (Fig. 6g). Again, we observed that cells with *Brcc3* inactivation were more sensitive to doxorubicin treatment as compared to cells with normal *Brcc3* status.

## Discussion

Here, we investigated the role of *BRCC3* mutations in the biology and treatment of t(8;21)(q22;q22.1) AML. In a large sequencing study of CBF AML we found recurrent inactivating mutations in *BRCC3* in t(8;21)(q22;q22.1) AML with a frequency of 4.7% but not in inv(16)(p13.1q22) AML [30]. Consistently, *BRCC3* mutations were found in 5 patients in a recent study of 331 t(8;21)(q22;q22.1) AML cases [38], but in only one patient with MDS-related cytogenetic abnormalities in the TCGA de novo AML cohort [29]. These results indicate a certain selectivity of *BRCC3* mutations for t(8;21)(q22;q22.1) AML. In our cohort, *BRCC3* mutations had no impact on preclinical characteristics but were associated with a favorable outcome with the caveat that the case number is small. Seven out of the nine *BRCC3* mutations were located within the catalytically active MPN + domain, four of them at the exact position as mutations previously found in MDS, while the other three were located in close proximity [28]. Given that most *BRCC3* mutations found in AML and MDS are predicted to be deleterious and are hemizygous we hypothesized that loss of BRCC3 function contributes to malignant transformation of hematopoietic cells in a specific genetic context.

In accordance with the close association of *BRCC3* mutations with t(8;21)(q22;q22.1) we found a pro-proliferative effect of *BRCC3* inactivation in the t(8;21)(q22;q22.1) AML cell lines Kasumi-1 and SKNO-1 but not in a cell line without t(8;21)(q22;q22.1). CRISPR-mediated *BRCC3* inactivation alone did not enhance the self-renewal capacity in mouse hematopoietic progenitor cells (LSK) implying that loss of *BRCC3* alone is not sufficient for malignant transformation. In contrast, self-renewal capacity was markedly increased in LSK cells when *BRCC3* was inactivated together with expression of *AML1-ETO* supporting that both genetic alterations cooperate.

We next sought to investigate how inactivation of *BRCC3* contributes to malignant transformation. One possible mechanism would be that loss of BRCC3 in the BRCA1-A complex leads to impaired DNA damage repair what may increase the likelihood for acquisition of genomic aberrations that activate oncogenic pathways. However, we considered this possibility for several reasons unlikely. First, in our AML t(8;21)(q22;q22.1) cohort no increase in secondary cytogenetic aberrations was observed and in MDS, *BRCC3* mutations are associated with a normal karyotype [28]. Second, the majority of the *BRCC3* mutations found in AML and also in MDS are subclonal, suggesting that they are a secondary event and not an initiating founder mutation that promotes further genetic alterations. Besides the role in DNA damage repair, BRCC3 is involved in other cellular pathways including cytokine signaling and regulation. BRCC3 as part of the BRISC-complex deubiquitinates its substrate IFNAR1 which affects interferon signaling [20]. We found that *BRCC3* mutations abrogate the DUB activity on IFNAR1 resulting in an attenuated interferon response in AML cells. In a study by DeKelver et al. it was shown that *AML1-ETO* induces strong interferon signaling and that this antagonizes the leukemic potential of *AML1-ETO* [36]. While *IFNAR1* has not been found mutated or deleted in t(8;21)(q22;q22.1) AML, we could show that *BRCC3* mutations render t(8;21)(q22;q22.1) AML cells less sensitive to IFNα-mediated toxicity through impaired deubiquitination and function of IFNAR1. This provides a possible explanation for the close association of *BRCC3* mutations with t(8;21)(q22;q22.1). We also demonstrate that AML cells with *BRCC3* inactivation had an impaired inflammasome activity with decreased IL-1β release after activation. In our models, IL-1β had no negative effect on cell proliferation of *AML1-ETO* cell lines. However, inflammasome-driven IL-1β has been found to suppress AML proliferation in other models [39]. Thus, downregulation of IL-1β by defective BRCC3 deubiquitination of NLRP3 could contribute to the transforming and proproliferative effects of *BRCC3* mutations.

In addition to IL-1β and interferon signaling, we found that *BRCC3* inactivation leads to deregulation of release of other cytokines by AML cells. Although not sufficient to induce cytokine-independence in several cell lines, some of the more released cytokines in *BRCC3* inactivated AML cells like G-CSF, HGF, and IL-4 have been shown to stimulate proliferation of AML cells [40–43]. We were able to directly tie increased G-CSF release to enhanced cell proliferation of t(8;21)(q22;q22.1) AML cells harboring *BRCC3* WT, but not to other cell lines with a different cellular background including inv(16)(p13.1q22), which further links *BRCC3* mutations to t(8;21)(q22;q22.1) AML. As loss of *BRCC3* on a subclonal level was sufficient to increase cell proliferation, this implies that *BRCC3* mutations at least in part act through a paracrine mechanism by release of G-CSF. A paracrine activation of AML cells would also explain how subclonal *BRCC3* mutations, as present in some of the AML and MDS patients, stimulate proliferation of nonmutated clones through pro-proliferative cytokines. Remarkably, primary AML cells and cell lines with t(8;21)(q22;q22.1) have a higher expression of G-CSF receptor molecules (G-CSFR) than other AML subtypes [41, 44]. G-CSF induces tyrosine phosphorylation of JAK2 through G-CSFR which leads to enhanced proliferation [45]. Of note, in a very recent study by Donaghy et al. BRCC3-mediated deubiquitination of JAK2 has been implicated in limiting hematopoietic stem cell expansion and knockdown of *BRCC3* was associated with an increased K63-ubiquitination and activation of JAK2 [46]. Therefore, *BRCC3* inactivation enhances JAK2 signaling by two mechanisms. Remarkably, JAK2 signaling is a particularly important driver in t(8;21)(q22;q22.1) AML as indicated by the presence of activating JAK2 mutations specifically in this but not other AML subtypes [47].

Enhanced self-renewal and proliferation by *BRCC3* mutations would suggest that they are associated with a more aggressive disease. However, the *BRCC3* mutated t(8;21)(q22;q22.1) AML patients in our cohort had an excellent outcome. In addition, in the TCGA dataset of de novo AML lower *BRCC3* expression was associated with a better overall survival [29]. Our observations in cell lines and murine primary hematopoietic cells suggest that *BRCC3* inactivation leads to an impaired capability of the BRCA1-A complex to repair DNA damage and subsequently higher sensitivity to DNA damaging chemotherapy. In fact, none of the *BRCC3* mutated patients in our cohort that received intensive chemotherapy relapsed. Consistently, downregulation of *BRCC3* and its complex members has been previously associated with a higher sensitivity to DNA damage inducing agents in other types of cancer [26, 37].

In conclusion, we demonstrate that *BRCC3* mutations lead to altered ubiquitination of its substrates and cytokine release which cooperates with *AML1-ETO* to induce AML and sensitizes the leukemic cells to cytotoxic chemotherapy. Future studies are needed to investigate the clinical impact of *BRCC3* mutations in larger cohorts in the context of different treatments and elucidate whether altered ubiquitination of additional, yet unknown substrates of BRCC3 contributes to the development of AML and MDS.

## Supplementary information

Supplement
