# Supplementary material for: Functional characterization of BRCC3 mutations in acute myeloid leukemia with t(8;21)(q22;q22.1)
Source: Leukemia. 2019 Oct 1;34(2):404–15. doi: 10.1038/s41375-019-0578-6 (PMC7214237; doi:10.1038/s41375-019-0578-6)
Supplement: Supplementary file 1 — Supplement [file 41375_2019_578_MOESM1_ESM.pdf]

Supplementary information to:

Functional characterization of *BRCC3* mutations in acute myeloid leukemia with t(8;21) (q22;q22.1)

Tatjana Meyer[1], Nikolaus Jahn[1], Stefanie Lindner[1], Linda Röhner[1], Anna Dolnik[2], Daniela Weber[1], Annika Scheffold[1], Simon Köpff[1], Peter Paschka[1], Verena I. Gaidzik[1], Dirk Heckl[3], Sebastian Wiese[1], Benjamin L. Ebert[4], Hartmut Döhner[1], Lars Bullinger[2], Konstanze Döhner[1], Jan Krönke[1]

[1]Department of Internal Medicine III, University Hospital of Ulm, Ulm, Germany; [2]Department of Hematology, Oncology, and Tumorimmunology, Charité University Medicine, Berlin, Germany; [3]Department of Pediatric Hematology and Oncology, Martin-Luther-University Halle-Wittenberg, Halle, Germany; [4]Department of Medical Oncology, Dana-Farber Cancer Institute, Boston, MA, USA

Corresponding author:

Jan Krönke, MD

Department of Internal Medicine III

University Hospital of Ulm

Albert-Einstein-Allee 23

89081 Ulm, Germany

Phone: +49-(0)731-500-45718

E-Mail: jan.kroenke@uni-ulm.de

Supplementary Materials and Methods

Patients and treatment

All patients were treated within a clinical trial of the German-Austrian AML Study Group (AMLSG; AML HD93, AML HD98A (NCT00146120), AMLSG 07-04 (NCT00151242), AMLSG 11-08 (NCT00850382), AMLSG 21-13 (NCT02013648)) or the AMLSG BiO Registry (NCT01252485). Of the 191 t(8;21)(q22;q22.1) AML patients, 187 received intensive chemotherapy, eighteen underwent allogeneic hematopoietic cell transplantation and two autologous transplantation in first remission. None of the *BRCC3* mutated patients underwent hematopoietic cell transplantation.

Library enrichment and sequencing

High throughput sequencing on the entire coding region of *BRCC3* was performed in 351 Core-binding factor AML patients [t(8;21)(q22;q22.1); n=191; inv(16)(p13.1q22), n=160][30]. For Library enrichment the commercially available SureSelectXT in-solution capture technology from Agilent Technologies (G9611B, Santa Clara, CA, USA) was used. Probe design was performed using Agilent's online tool SureDesign. The USCS human genome 19 (H. sapiens, hg19, GRCh37, February 2009) served as reference genome for design and determination of genomic coordinates.

Genomic DNA (200 ng per sample) extracted from diagnostic bone marrow (81%) or peripheral blood (19%) specimens was used for molecular screening. SureSelect library preparation and indexing were performed following the manufacturer's instructions for Illumina paired-end sequencing. Samples were then transferred to a cBot (Illumina, San Diego, CA, USA) to create clonal clusters on a flow cell by bridge amplification (Illumina reagent kit: TruSeq PE Cluster Kit v3-cBot-HS). Finally, 2x 100 bp paired-end sequencing by synthesis was carried out on a HiSeq2000 (Illumina) using Illumina's TruSeq SBS Kit v3-HS reagents, yielding a median *BRCC3* on-target sequencing depth of 1571x (range 718 - 4607).

Variant calling

The sequencing quality of each sample was assessed using the NGS QC toolkit (2.3.3) and, where necessary, adapter and read end trimming were performed using cutadapt (1.8.3) and in-house scripting respectively.

Paired-end reads were then aligned to the hg19 reference using BWA-MEM (0.7.10). Alignments are sorted and indexed by Picard (1 .138) and locally realigned using GATK (3.4.46). For each sample, coverage statistics were calculated using BEDTools (2.24.0) and processed by SAMtools (0.1.19). VarScan2 (2.3.9) was then used for variant calling within the target regions sequenced. All variants were annotated by Annovar (release 22Mar2015) but only non-synonymous mutations affecting exons or splice sites were retained. These were further filtered to remove calls within known regions of segmental duplication, variants annotated in dbSNP (138) but not COSMIC (70) and variants with a minor allele frequency (MAF) above 0.01 in either the 1000 Genomes Project or the Exome Sequencing Project (ESP 6500).

Cell culture

Cell lines THP-1, OCI-AML5, Kasumi-1, HEK-293T, 32D, and SKNO-1 were obtained from the American Type Culture Collection (ATCC) or the German Collection of Microorganisms and Cell Culture (DSMZ). All cell lines were authenticated using STR-profiling and tested for mycoplasma contamination. THP-1 and OCI-AML5 cell lines were grown in RPMI 1640 medium (F1215, Merck Millipore, Burlington, MA, USA) supplemented with 10% Fetal Bovine Serum (FBS) (S 0615, Merck Millipore), 1% Penicillin-Streptomycin (15140122, Thermo Fisher Scientific, Waltham, MA, USA), and 1% L-Glutamine (K 0282, Merck Millipore). The Kasumi-1 cell line was cultured in RPMI 1640 supplemented with 20% FBS, 1% Penicillin-Streptomycin, and 1% L-Glutamine from the same suppliers. The SKNO-1 cell line was grown in RPMI 1640 medium supplemented with 10% FBS, 1% Penicillin-Streptomycin, 1% L-Glutamine, and 10 ng/mL recombinant human GM-CSF (300-03, PeproTech Rocky Hill, NJ, USA. The HEK-293T

cell line was cultured using Dulbecco's Modified Eagle Medium (DMEM) (11995065, Thermo Fisher Scientific) supplemented with 10% FBS, 1% Penicillin-Streptomycin, and 1% L-Glutamine. The 32D cell line was grown in RPMI 1640 medium supplemented with 10% FBS, 1% Penicillin-Streptomycin, 1% L-Glutamine, and 10 ng/ml recombinant murine interleukin-3 (IL-3) (213-13, PeproTech). All cells were cultured at appropriate densities according to their growth requirements and split every 2-3 days.

Growth curves

For growth curve analysis, 1 x 10^6 Kasumi-1, SKNO-1 and OCI-AML5 cells containing either *BRCC3*^{WT} or *BRCC3*^{KO} were seeded in triplicates. To evaluate the growth of *BRCC3* mutations, Kasumi-1 cells harboring a *BRCC3*^{KO} were retrovirally transduced with either pRSF91-MCS-IRES-GFP-T2A-BRCC3-R81X-SM or pRSF91-MCS-IRES-GFP-T2A-BRCC3-R81G-SM. Next, 5 x 10^5 Kasumi-1 cells containing either *BRCC3*^{WT}, *BRCC3*^{R81X}, or *BRCC3*^{R81G} were seeded in triplicates. To assess the impact of sub-clonal *BRCC3*^{KO}, Kasumi-1 cells stably expressing the Cas9 enzyme were transduced at a ratio of approximately 40% with pLKO5.hU6.sgRNA.dTom.BRCC3.ex1 or pLKO5.hU6.sgRNA.dTom.Luciferase. Then, 5 x 10^5 Kasumi-1 cells containing either 40% *BRCC3*^{KO} or 40% of a non-targeting sgRNA control were seeded in triplicates. All cells were counted at the indicated time points and the presence of pLKO5.hU6.sgRNA.dTom BRCC3 ex1 or pLKO5.hU6.sgRNA.dTom Luciferase was monitored by flow cytometry. Cells were split every 2 - 3 days and refilled with fresh medium to ensure exponential growth. To assess growth with exogenous G-CSF, cells were cultured with 10 ng/ml recombinant G-CSF (300-23, PeproTech). To inhibit G-CSF, 10 ng/ml of a neutralizing anti-G-CSF antibody (MAB214-100, R&D systems, Minneapolis, MN, USA) was used. To analyze the impact of exogenous IL-1β or IFNα, 10 ng/ml IL-1β (200-01B, PeproTech) or IFNα (SRP4596, Sigma-Aldrich, St. Louis, MO, USA) were utilized.

Cytokine analyses

To assess IL-1β secretion in THP-1 *BRCC3* WT, R81X, R81G, and KO cells, THP-1 cells were seeded at 1 x 10^6 cells/ml and stimulated with 100 nM PMA (P1585, Sigma-Aldrich) for 48 hours. Then, cells were washed twice in sterile PBS and cultured for 48 hours in PMA-free media. Cells were then stimulated with 100 ng/ml LPS (L6143, Sigma-Aldrich) for 16 hours followed by stimulation with 2 mM ATP (P0756L, New England Biolabs) for 30 minutes. Supernatants were collected and the Human IL-1β/IL-1F2 Quantikine ELISA Kit (DLB50, R&D Systems, Minneapolis, MN, USA) used according to the manufacturer's protocol.

Cytokine array was performed as recommended by the protocol: 1.5 x 10^6 cells of Kasumi-1 *BRCC3* WT or KO were washed twice with sterile PBS and seeded in 2 ml media overnight. Then, the supernatants were collected and the cytokine array (ab133998, Abcam) carried out according to the manufacturer's instructions.

Cytokine-independent growth in 32D and SKNO-1 cells

To analyze the effect of *BRCC3* or *Brcc3* knock-out on cytokine-independent growth, 32D cells stably expressing the Cas9 enzyme were infected with multiple sgRNA vectors (pLKO5.hU6.sgRNA.dTom) against *Brcc3*, a non-targeting sgRNA control or a vector containing $Kras^{G13D}$ (pRSF91-MCS-IRES-GFP-T2A-Puro). In a similar manner, SKNO-1 cells stably expressing the Cas9 enzyme were transduced with two sgRNA vectors targeting *BRCC3* or a non-targeting control sgRNA. The cells were cultured in medium supplemented with 10 ng/ml recombinant murine interleukin-3 (IL-3) for 32D cells or 10 ng/ml recombinant GM-CSF for SKNO-1 cells for seven days to ensure proper genomic cleavage. The sgRNA-induced cleavage was confirmed using the GeneArt Genomic Cleavage Kit (A24372, Thermo Fisher Scientific). Then, the cells were washed twice in sterile DPBS buffer (14200067, Thermo Fisher Scientific) and cultured in medium without supplement of cytokines. The presence of cells containing the transduced plasmids was monitored using flow cytometry at the indicated time points.

Colony forming unit (CFU) assay

Murine stem- and progenitor cells (LSK) from the B6J. 129(Cg) -Gt(ROSA)26Sor tm1.1(CAG-cas9*,-EGFP)Fezh/J strand (#026179, Jackson Laboratory)[35] stably expressing the Cas9 enzyme were sorted via fluorescently-activated cell sorting (FACS) using the following antibodies manufactured by BioLegend: Pacific Blue™ anti-mouse Lineage Cocktail (133310), PE anti-mouse Ly-6A/E (Sca-1) (108108), and APC anti-mouse CD117 (c-Kit) (105812). Afterwards, the cells were infected with both a lentivirus (pLKO5.hU6.sgRNA.dTom) containing either a pool of three sgRNAs targeting *Brcc3* or a control as well as with a retrovirus containing either AML1-ETO or a control (pMY-IRES-GFP-AML1/ETO or pMY-IRES-GFP). All vectors were fluorescently labeled. Cells (1 x 10^3) were seeded in methylcellulose without supplement of cytokines (MethoCult GF M3434, StemCell Technologies, Vancouver, Canada). Colonies were counted after 10-14 days and pictures were taken using a Nikon Digital Sight DS-U1 camera (Minato, Japan) attached to a Nikon Eclipse TS100 microscope. Then, the colonies were detached using CliniMACS® PBS/EDTA buffer (Miltenyi Biotec, Bergisch Gladbach, Germany) and 1 x 10^4 cells were seeded for replating. The remaining cells were analyzed using flow-cytometry and May-Grünwald Giemsa staining. To analyze the effect of G-CSF on colony formation, LSK cells transduced with either *AML1-ETO* or *AML1-ETO* and multiple sgRNA vectors targeting *Brcc3* were cultured in the presence of either 10 ng/ml murine G-CSF (250-05, PeproTech) or a vehicle control. The number of colonies was determined as the number of colonies positive for both transduced vectors.

Co-Immunoprecipitation

4 x 10^5 HEK-293T cells were seeded per well and transfected them with 1000 ng of DNA of FLAG-tagged *BRCC3* WT or mutants. 48h post transfection, cells were lysed in ice cold NP40-buffer (FNN0021, Thermo Fisher Scientific) supplemented with 1x Halt™ Protease and Phosphatase Inhibitor Single-Use Cocktail (78443, Thermo Fisher Scientific), incubated on ice for 10 min and centrifuged for 10 min at 16 100 g and 4°C. Then, the supernatant was incubated with overnight with magnetic FLAG-beads (M8823, Sigma-Aldrich). After washing

the beads twice in NP40-buffer supplemented with 1x Halt™ Protease and Phosphatase Inhibitor Single-Use Cocktail, proteins were eluted for 30 min at 4°C using FLAG-peptide (F3290, Sigma-Aldrich). The analysis was performed through western blot.

RNA-extraction and RT-PCR

1 x 10^6 cells of Kasumi-1 *BRCC3* WT or KO were washed with sterile PBS and seeded in 2 ml media overnight. Following activation with 500 U/ml IFNα (SRP4596, Sigma-Aldrich) for 0 and 60 min, RNA was isolated using the RNeasy Mini Kit (74104), the QIAshredder (79654, both by Qiagen), cDNA was transcribed using the SuperScript II Reverse Transcriptase (18064014, Invitrogen, Carlsbad, CA, USA) and the qRT-PCR was performed using the SYBR Green PCR Kit (204074, Qiagen), all according to the manufacturer's protocol. All primers used for the qRT-PCR have been previously described[36].

Ubiquitination analyses

HEK-293T cells containing a *BRCC3* knock-out were seeded at a density of 4 x 10^5 cells per well and transfected with 1000 ng of DNA of multiple plasmids containing FLAG-tagged *interferon (IFN) receptor chain 1* (*IFNAR1*), HA-tagged *K63-Ubiquitin* and wild type or mutated *BRCC3*. 48 hours post infection, the cells were lysed in denaturing buffer (2% SDS, 150 mM NaCl, 10 mM Tris, pH = 8.0) and heated to 95°C for 10 min, then ice cold NP40-buffer was added supplemented with 1x Halt™ Protease and Phosphatase Inhibitor Single-Use Cocktail, 10 µM MG-132 (S2619, SelleckChem), and 50 nM PR619 (S7130, SelleckChem) and all samples were passed through a 20G syringe multiple times. The samples were incubated on ice for 10 min and centrifuged for 10 min at 16 100 g and 4°C. Then, samples were incubated overnight with magnetic FLAG-beads. After washing the beads twice in NP40-buffer supplemented with MG-132 and PR619, proteins were eluted for 30 min at 4°C using FLAG-peptide. The analysis was performed by western blot.

Mass spectrometric analysis

To analyse interaction partners of BRCC3 wild type (WT) or mutants, we applied stable isotope labelling of amino acids in cell culture (SILAC)-based quantitative mass spectrometry[1]. A co-immunoprecipitation was performed in OCI-AML5 stably over-expressing an empty control, FLAG-tagged BRCC3 WT, BRCC3 R81G, or BRCC3 D135E. The samples were then separated by SDS-PAGE on 12 % self-made Bis-Tris gels. The gel was stained by colloidal coomassie staining by pre-incubating in staining solution (34 % (v/v) methanol, 2 % (v/v) phosphoric acid, 17 % (w/v) ammonium sulfate) for 1 h at RT and subsequent addition of 0.066 % (w/v) Coomassie brilliant blue G-250. After incubation overnight, the gel was de-stained with ddH2O.

Following trypsin digestion, peptides eluted from de-stained gel slices were subjected to mass spectrometric analysis using an LTQ Orbitrap Velos Pro system (Thermo Fisher Scientific) online coupled to an U3000 RSLCnano (Thermo Fisher Scientific) as described previously[2] with the following exceptions: A binary solvent gradient consisting of solvent A (0.1 % FA) and solvent B (86 % ACN, 0.1 % FA) was used for separation. The column was initially equilibrated in 5 % B. In a first elution step, the percentage of B was raised from 5 % to 15 % in 5 min, followed by an increase from 15 % to 40 % B in 30 min. The column was washed with 95 % B for 4 min and re-equilibrated with 5 % B for 25 min.

Using the embedded Andromeda search engine[3] within MaxQuant Vers. 1.5.2.8[4], MS/MS spectra were correlated with the UniProt human reference proteome set. The respective SILAC amino acids, Arg6 + Lys4 and Arg10+Lys8 were selected, while Carbamidomethylated cysteine was considered as a fixed modification along with oxidation (M), and acetylated protein N-termini as variable modifications. False discovery rates were set on both, peptide and protein level, to 0.01. Proteins having fold changes in both replicates below -2 or above 2 were considered as specifically regulated.

Supplementary Figures

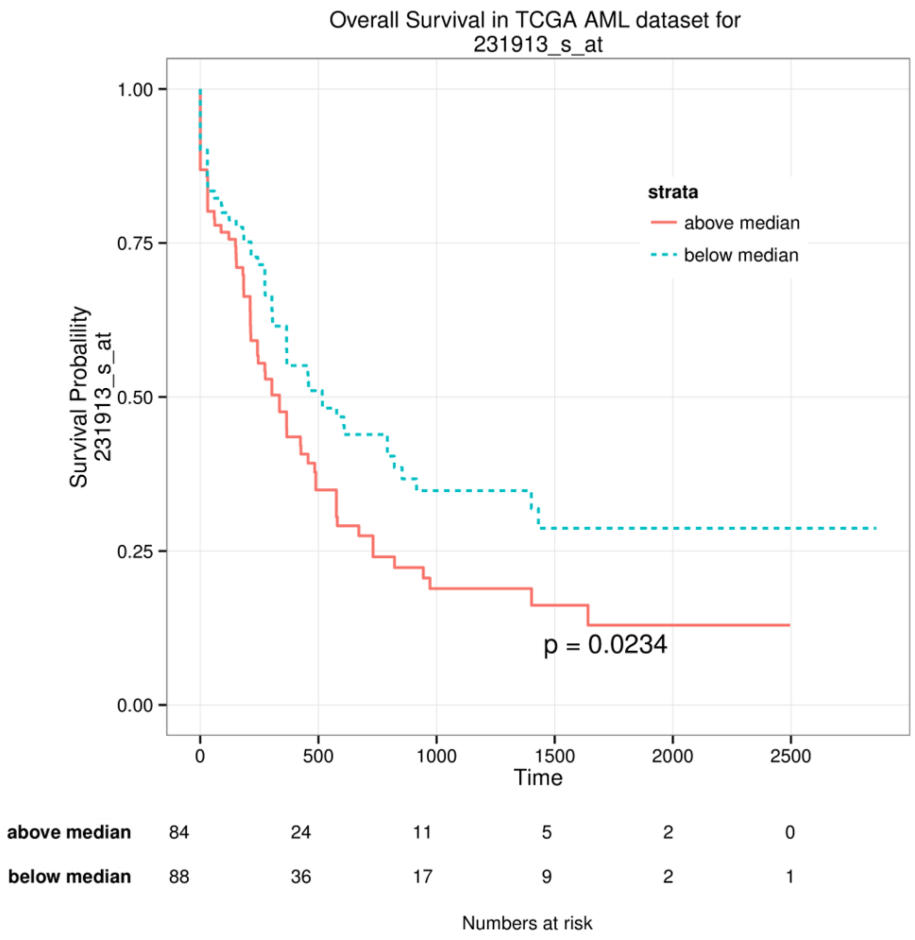

Supplemental Figure 1: Overall survival of AML patients expressing different levels of *BRCC3*. Data was taken from the TCGA AML database[29] and analyzed by BloodSpot[34].

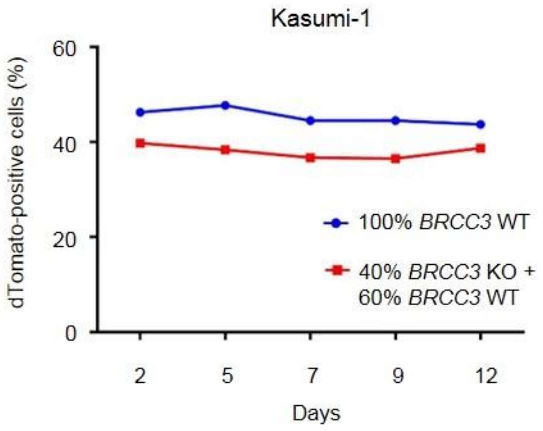

Supplemental Figure 2: Transduction rate of sub-clonal *BRCC3* KO Kasumi-1 cells. Kasumi-1 cells were transduced with either a sgRNA targeting *BRCC3* or a non-targeting control and the transduction rate was assessed by flow cytometry at the indicated times.

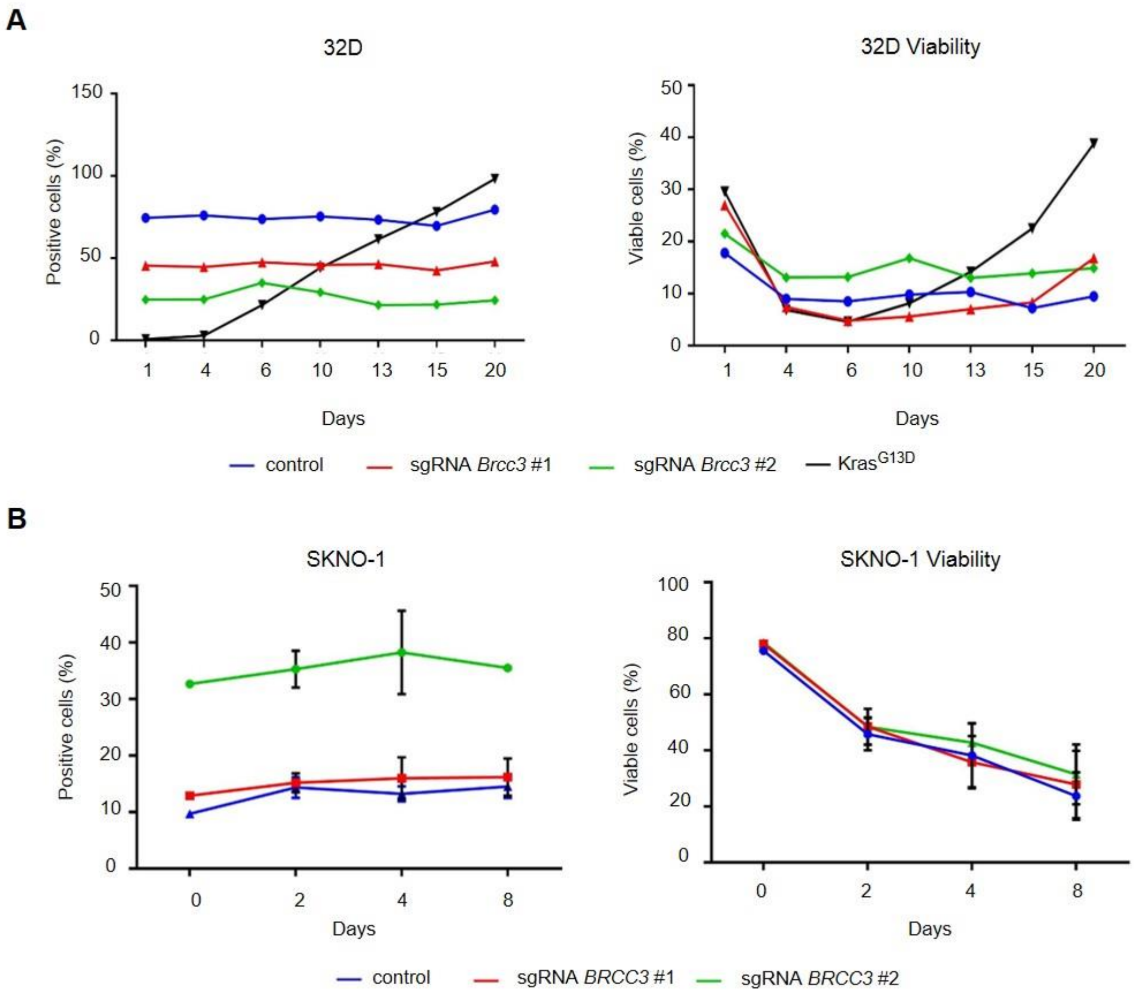

Supplemental Figure 3: Cytokine-independent growth in murine *Brcc3* WT and KO 32D and human *BRCC3* WT and KO SKNO-1 cell lines. The percentage of transduced, GFP- or dTomato-positive vectors as well as cell viability were monitored in 32D **(a)** and SKNO-1 cells **(b)** using flow cytometry. Results shown are representative for 2 independent experiments.

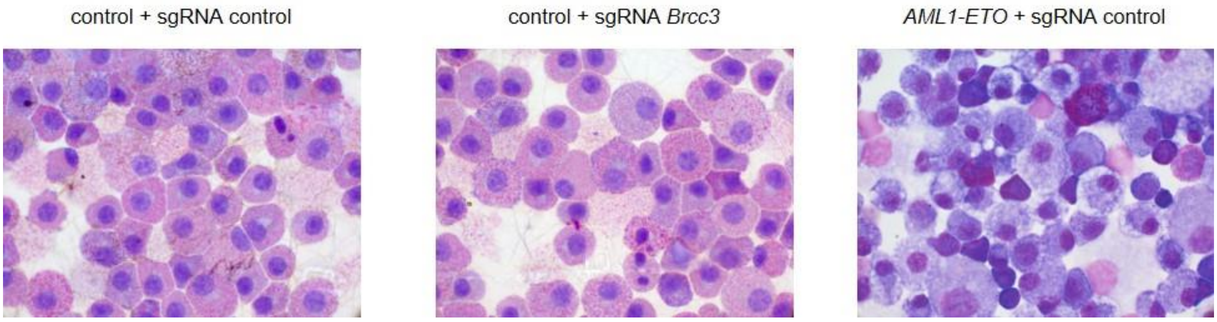

Supplemental Figure 4: Microscopic assessment of LSK cells. LSK cells transduced with either two control vectors, one control vector and multiple sgRNA vectors targeting *Brcc3*, or *AML1-ETO* and a control sgRNA were assessed by May-Grünwald staining.

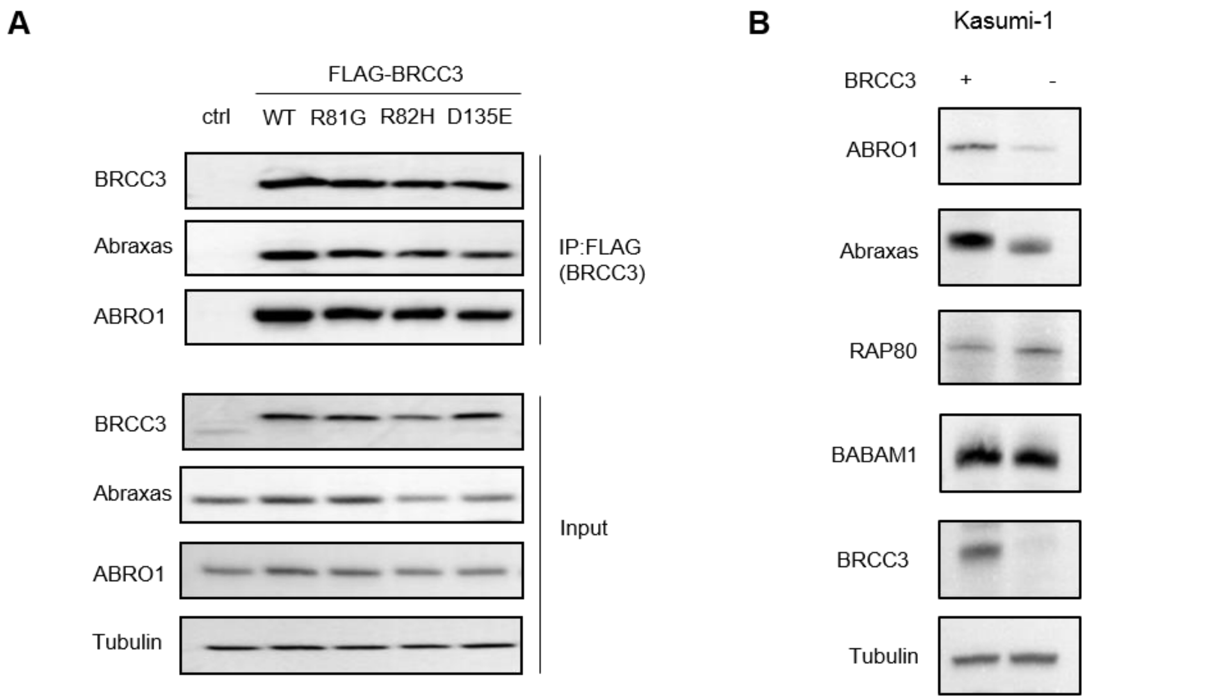

Supplemental Figure 5: Impact of BRCC3 WT, mutants, and KO on BRCA1-A and BRISC complex formation. a Immunoprecipitation of FLAG-tagged BRCC3 WT or mutants in HEK-293T cells. **b** Effect of *BRCC3* KO in Kasumi-1 cells on the protein level of BRCA1-A and BRISC complex members.

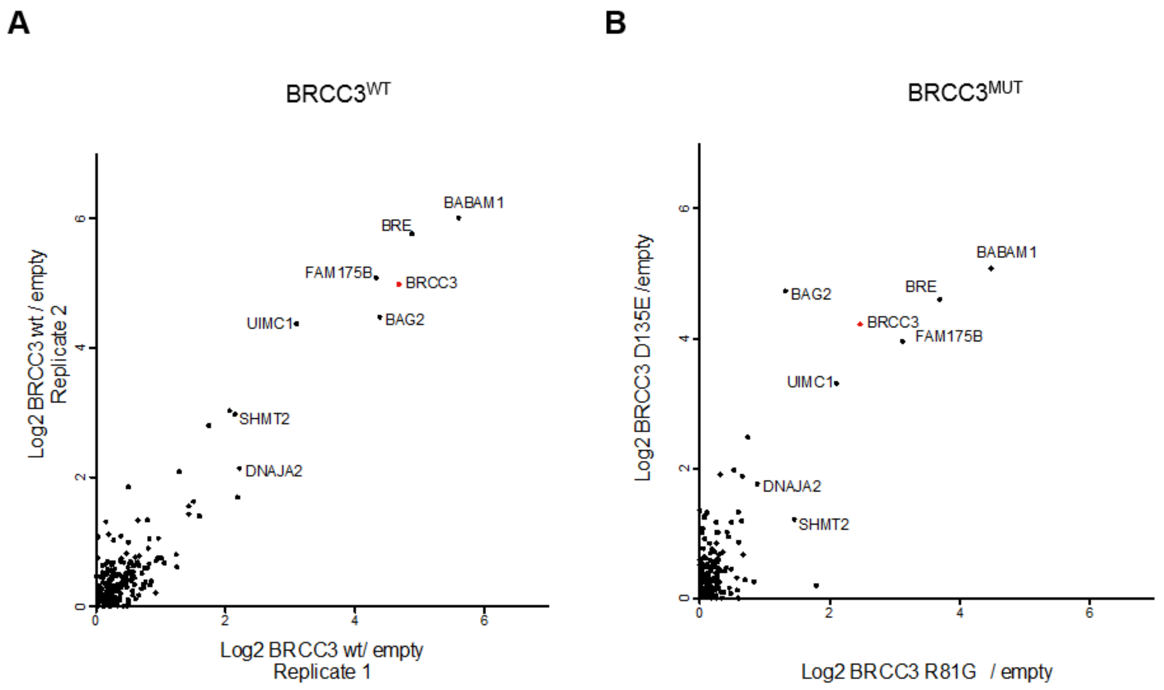

C

	Median Log(2) ratio	
**Protein**	**BRCC3^WT^**	**BRCC3^MUT^**
BABAM1/MERIT40	5.80	4.78
BRE/BRCC45	5.32	4.15
Abraxas/FAM175A	5.07	3.65
BRCC3	4.83	3.34
ABRO1/FAM175B	4.71	3.54
BAG2	4.43	3.02
UIMC1/RAP80	3.74	2.71
SHMT2	2.56	1.33
HSPA8	2.55	1.61
HSPA1A	2.27	1.27

Supplemental Figure 6: SILAC-based proteomic analysis of FLAG-immunoprecipitation of BRCC3 WT, R81G, and D135E. Quantitative SILAC-based mass spectrometry was performed after FLAG-immunoprecipitation of FLAG-tagged BRCC3 WT **(a)**, BRCC3^{R81G}, and BRCC3^{D135E} **(b)** in OCI-AML5 cells. The experiment was set up in independent biological replicates. **c** Top hits for interaction partners of BRCC3 WT and mutants visualized as the median log(2) ratio of protein abundance bound to FLAG-BRCC3 vs empty control.

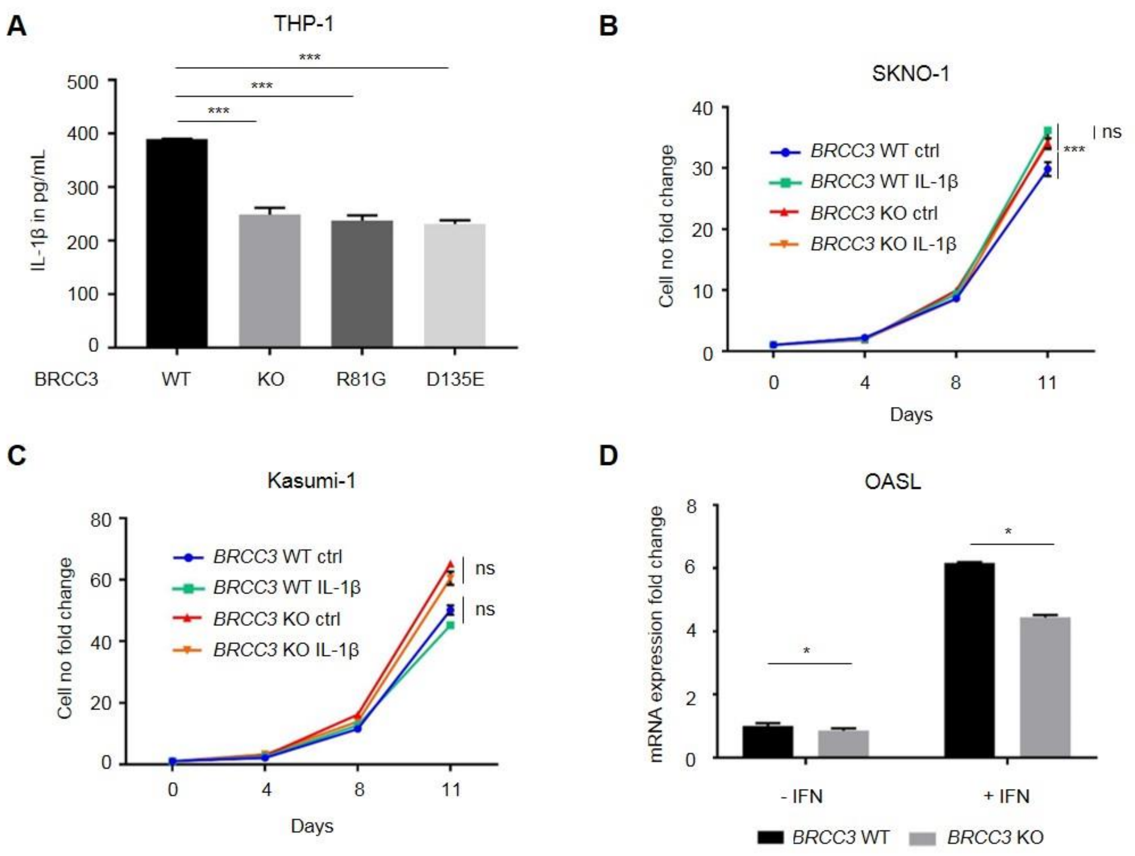

Supplemental Figure 7: Effect of *BRCC* KO on IL-1β and IFN-response gene *OASL*. a IL-1β release of THP-1 cells containing either *BRCC3* WT, *BRCC3* R81G, D135E, or KO as measured by ELISA. Cell proliferation of **(b)** SKNO-1 and **(c)** Kasumi-1 *BRCC3* WT and *BRCC3* KO cells treated with exogenous IL-1β for 11 days. **d** mRNA-expression of *OASL* in *BRCC3* WT or KO Kasumi-1 cells upon IFN-stimulation. *P < 0.05, **P < 0.01, ***P < 0.001. All p-values are calculated using unpaired Student *t* test. Error bars are standard error of the mean. n = 3 for data shown, which is representative of 3 independent experiments.

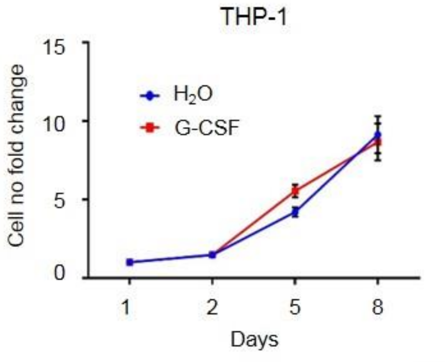

Supplemental figure 8: Proliferation of THP-1 cells in the presence of G-CSF. *BRCC3* WT THP-1 cells were treated with G-CSF and cell proliferation was assessed at the indicated time points. Data is representative of 3 independent experiments.

Supplementary references

1. Ong S-E, Blagoev B, Kratchmarova I, Kristensen DB, Steen H, Pandey A, u. a. Stable isotope labeling by amino acids in cell culture, SILAC, as a simple and accurate approach to expression proteomics. Mol Cell Proteomics. Mai 2002;1(5):376–86.

2. Mohr KB, Zirafi O, Hennies M, Wiese S, Kirchhoff F, Münch J. Sandwich enzyme-linked immunosorbent assay for the quantification of human serum albumin fragment 408–423 in bodily fluids. Analytical Biochemistry. Mai 2015;476:29–35.

3. Cox J, Neuhauser N, Michalski A, Scheltema RA, Olsen JV, Mann M. Andromeda: A Peptide Search Engine Integrated into the MaxQuant Environment. Journal of Proteome Research. April 2011;10(4):1794–805.

4. Cox J, Mann M. MaxQuant enables high peptide identification rates, individualized p.p.b.-range mass accuracies and proteome-wide protein quantification. Nature Biotechnology. Dezember 2008;26(12):1367–72.
